# Disrupted Circadian Rhythms in a Mouse Model of Schizophrenia

**DOI:** 10.1016/j.cub.2011.12.051

**Published:** 2012-02-21

**Authors:** Peter L. Oliver, Melanie V. Sobczyk, Elizabeth S. Maywood, Benjamin Edwards, Sheena Lee, Achilleas Livieratos, Henrik Oster, Rachel Butler, Sofia I.H. Godinho, Katharina Wulff, Stuart N. Peirson, Simon P. Fisher, Johanna E. Chesham, Janice W. Smith, Michael H. Hastings, Kay E. Davies, Russell G. Foster

**Affiliations:** 1 MRC Functional Genomics Unit, Department of Physiology, Anatomy and Genetics, University of Oxford, Parks Road, Oxford OX1 3PT, UK; 2 Nuffield Laboratory of Ophthalmology, University of Oxford, Levels 5 and 6 West Wing, John Radcliffe Hospital, Headley Way, Oxford OX3 9DU, UK; 3 Division of Neurobiology, MRC Laboratory of Molecular Biology, Hills Road, Cambridge CB2 0QH, UK; 4 Circadian Rhythms Group, Max Planck Institute for Biophysical Chemistry, Am Fassberg 11, 37077 Gottingen, Germany; 5 Lilly, Erl Wood Manor, Sunninghill Road, Windlesham, Surrey GU20 6PH, UK

## Abstract

Sleep and circadian rhythm disruption has been widely observed in neuropsychiatric disorders including schizophrenia [1] and often precedes related symptoms [2]. However, mechanistic basis for this association remains unknown. Therefore, we investigated the circadian phenotype of *blind-drunk* (*Bdr*), a mouse model of synaptosomal-associated protein (Snap)-25 exocytotic disruption that displays schizophrenic endophenotypes modulated by prenatal factors and reversible by antipsychotic treatment [3, 4]. Notably, *SNAP-25* has been implicated in schizophrenia from genetic [5–8], pathological [9–13], and functional studies [14–16]. We show here that the rest and activity rhythms of *Bdr* mice are phase advanced and fragmented under a light/dark cycle, reminiscent of the disturbed sleep patterns observed in schizophrenia. Retinal inputs appear normal in mutants, and clock gene rhythms within the suprachiasmatic nucleus (SCN) are normally phased both in vitro and in vivo. However, the 24 hr rhythms of arginine vasopressin within the SCN and plasma corticosterone are both markedly phase advanced in *Bdr* mice. We suggest that the *Bdr* circadian phenotype arises from a disruption of synaptic connectivity within the SCN that alters critical output signals. Collectively, our data provide a link between disruption of circadian activity cycles and synaptic dysfunction in a model of neuropsychiatric disease.

## Graphical abstract

**Graphical Abstract:**

**Figure:**
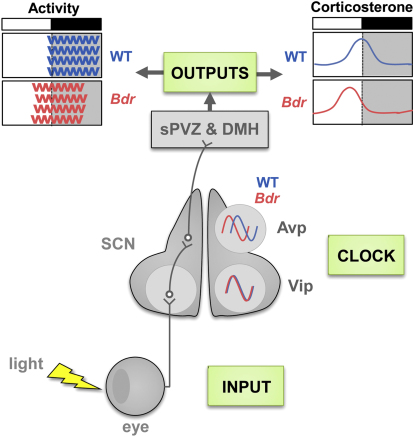

**Highlights:**

► A mouse Snap-25 mutant shows abnormal circadian behavior reminiscent of schizophrenia ► The phase advance of activity is mirrored by SCN output and corticosterone release ► Importantly, the core molecular clock of the Snap-25 mutant is not affected ► We discuss a link between neurotransmission, circadian defects, and schizophrenia

## Results

**Figure 1:**
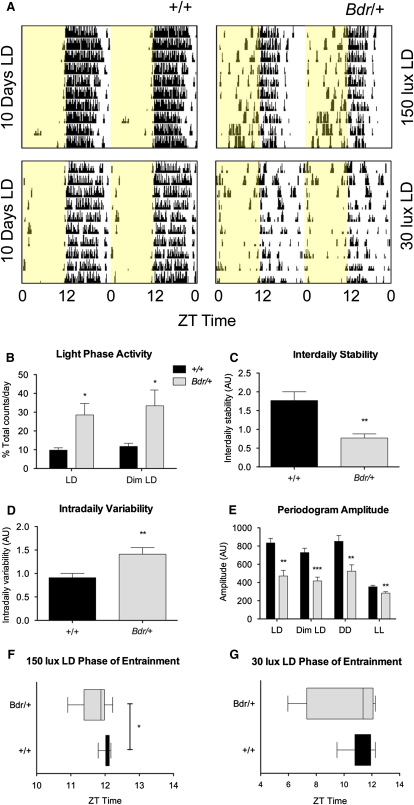
*Bdr* Mutants Display a Range of Circadian Abnormalities (A) Representative actograms of mouse running-wheel activity counts (black bars displayed in 10 min bins) show increased light activity (yellow panels) with less consistent and shorter major activity periods in *Bdr* compared to controls under both 150 and 30 lux 12 hr LD cycles. (B) Based on wheel-running behavioral data, *Bdr (Bdr/+)* mutants show increased light activity in LD and dim LD compared to wild-type controls (+/+) (n = 9–12; p values: LD = 0.0132, dim LD = 0.0226, analysis of variance [ANOVA], planned comparison). (C–G) LD rhythmicity showed that interdaily stability (C) was also significantly reduced in *Bdr* animals (n = 11–12; p = 0.0011, t test) and intradaily variability (D) was significantly increased in Bdr animals compared to controls (n = 11–12; p = 0.0065, t test). Overall periodogram amplitude was reduced in mutants under all lighting conditions (E) (n = 8–12; p values: LD = 0.0027, dim LD = 0.0004, constant darkness [DD] = 0.0061, and lux constant light [LL] = 0.0075, ANOVA). Across both LD conditions, entrainment is highly variable in the *Bdr* cohort (F and G) and significantly earlier in the *Bdr* animals under 150 lux LD (F) (n = 10–12, p = 0.0269, ANOVA). Data are presented as mean ± SEM. The full data set and p values are summarized in Table S2.

Circadian wheel-running behavior in *blind-drunk* (*Bdr*) mutants is markedly abnormal under light/dark (LD) conditions; actograms showed an advanced circadian phasing, fragmentation, and poor consolidation of nocturnal activity (Figure 1A). Total activity levels of the *Bdr* mice were reduced compared to controls, and this can be attributed to the mild ataxia seen in these animals (see Figure S1A available online). However, there was a significant increase in light phase activity (19% at 150 lux and 22% at 30 lux) during a 12 hr light/12 hr dark (12:12 LD) schedule (Figure 1B). Reduced stability and increased variability (Figures 1C and 1D) appear to underpin circadian rhythm fragmentation and instability in *Bdr* mice maintained under an LD cycle. Activity fragmentation is demonstrated further in mutants by more frequent activity bouts under LD (Figure S1B) with a decrease in periodogram amplitude compared to littermates (Figure 1E). In addition, the phase of entrainment was significantly advanced by 21.54 ± 7.92 min under a 150 lux LD cycle and particularly variable under 30 lux dim LD in *Bdr* mice compared to controls (Figures 1F and 1G). Heterogeneity in the locomotor behavior of *Bdr* mice studied under a 12:12 LD cycle was marked, with activity ranging from robust rhythms and entrained to highly irregular profiles (Figure S1D). This range of circadian phenotypes is similar to the heterogeneity observed in the circadian rhythms of neuropsychiatric illness. Specifically, abnormal phasing to the light/dark cycle, rest and activity fragmentation, and circadian reversal have been reported in schizophrenia [1, 17–20]; allowing for nocturnal versus diurnal activity patterns, there are some notable parallels between the *Bdr* and human circadian phenotypes as shown (Figure S2).

**Figure 2:**
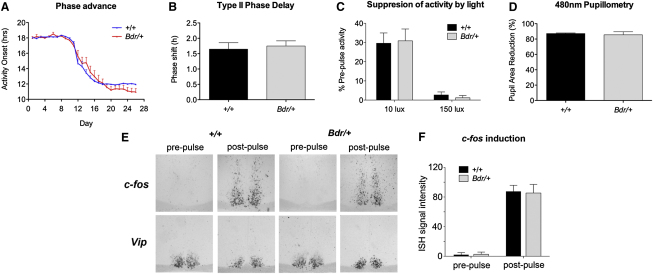
The Behavioral and Physiological Response to Light Is Not Altered in *Bdr* Mice (A–D) The ability of the circadian activity rhythm to phase shift following a 6 hr phase advance (A) and an acute photic stimulus in the respective night (B) are comparable in *Bdr* mice and wild-type controls. The ability for acute suppression of activity (negative masking) by light (C) and the 480 nm light-driven reduction in pupil area (D) (correlate of melanopsin-driven pupil constriction) are comparable in *Bdr* mice and wild-type controls. No significant effect of genotype is observed with any parameter presented. See Table S2 for related p values. (E and F) Representative ISH of *c-fos* and *Vip* in the mid-SCN both before and after a 30 min light pulse (E). Quantification of *c-fos* expression shows equivalent levels of light induction in both genotypes (n = 3–4) (F). Data are presented as mean ± SEM.

To determine whether abnormalities in the light input to the clock are modulating the *Bdr* phenotype, we exposed mice to an acute phase shifting light pulse and a 6 hr LD cycle phase advance (Figure S1C). The resulting phase shifting effects were not significantly different between genotypes (Figures 2A and 2B). In addition, negative masking in *Bdr* mice was comparable to controls (Figure 2C), suggesting that abnormalities in the suppression of activity by light cannot account for the *Bdr* phenotype. No differences were identified in gross retinal anatomy (data not shown) or light-driven pupil constriction in *Bdr* mice (Figure 2D) suggesting that abnormal inputs from the eye are not the source of the circadian phenotype. We also measured light induction of the immediate early gene *c-fos*, and equivalent levels of expression were seen in both *Bdr* and wild-type suprachiasmatic nucleus (SCN) sections (Figures 2E and 2F), providing further evidence that photic input to the SCN is not disrupted in mutant animals. A summary of the behavioral screen parameters and the complete set of circadian data are shown in Tables S1 and S2.

To address whether the circadian phenotype of the *Bdr* mutant was an artifact of using running wheels, we undertook 24 hr home cage video tracking of *Bdr* and wild-type mice [21]. These data replicate the advanced phase of activity and the negative masking previously observed by wheel running (Figures S3A–S3E). Additionally, estimations of total sleep duration [21] were not significantly different between genotypes (Figure S3F).

**Figure 3:**
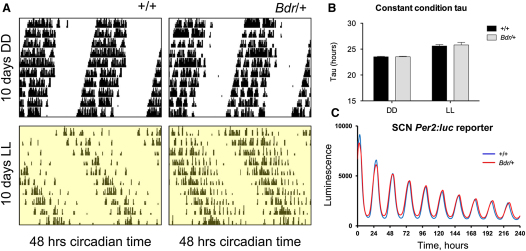
The Core Molecular Clock Is Not Altered in *Bdr* Mice (A) Representative actograms of mouse running-wheel activity counts (black bars displayed in 10 min bins) show comparable free-running rhythmicity and activity patterns in *Bdr* compared to controls under both DD and 150 LL cycles. Increased activity fragmentation compared to wild-types is observed in the *Bdr* mice; this is reflected in periodogram amplitude values for both DD and LL (Figure 1). (B) The free-running period or tau (τ) in both DD and LL shows no significant difference (ANOVA, planned comparison) between *Bdr* and wild-type mice as a marker of core clock stability, data summarized in Table S2. Data are presented as mean ± SEM. (C) Representative bioluminescence of organotypic SCN slice culture from the *Per2:Luc* reporter line either wild-type (blue) or *Bdr* mutant (red).

To establish whether the *Bdr* phenotype might be related to disruption of the core molecular clock, we undertook both in vivo and in vitro approaches. Under constant conditions, actograms were broadly similar and free-running circadian periods (*tau*) were not significantly altered between genotypes (Figures 3A and 3B). SCN molecular rhythms were then examined in detail by crossing *Bdr* mice with a *Per2:Luciferase* reporter strain. Consistent with the behavioral findings under constant conditions, we observed no significant differences in *Per:Luc* bioluminescence rhythms in the SCN when compared to wild-type controls (Figure 3C) or in recordings from the prefrontal cortex and cerebellum (data not shown).

The data show that *Bdr* mice lack the ability to maintain robust and appropriately phased activity rhythms under LD cycles, whereas their circadian locomotor behavior under constant conditions is comparable to wild-type littermates. Both retinal function and the molecular clock appear normal. Collectively, these observations suggested that the abnormal phenotype of *Bdr* mice might be due to defects in SCN outputs and the ability of the SCN to regulate peripheral oscillations. To address this issue, we undertook microarray expression profiling from SCN-enriched tissue punches across a 150 lux LD cycle from *Bdr* and wild-type mice. Gene expression was assessed during the mid-light phase (ZT6), the late light phase (ZT11), and the early dark phase (ZT13) when the most profound differences in locomotor activity were observed between genotypes. The accuracy of tissue punches was validated using *Six6* as a neuroanatomical marker of the SCN (Figure S4A). In view of the importance of Snap-25 in neurotransmission, surprisingly few differences in gene expression were observed between genotypes (Table S3). However, a number of key genes did show highly significant temporal changes in *Bdr* mice when compared to wild-types, including the SCN neuropeptides arginine vasopressin (*Avp*), neurotensin (*Nts*), and tachychinin 1/substance P (*Tac1*/*Sp*) (Table S4), all genes whose protein products have been variously implicated as SCN output signals but whose specific function is unclear [22, 23].

**Figure 4:**
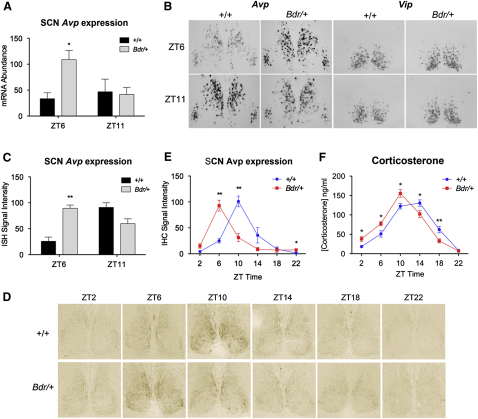
Phase Advances of Avp Expression in the SCN and Serum Corticosterone Occurs in *Bdr* Mice (A) qRT-PCR from SCN-enriched tissue punches shows advanced peak of *Avp* expression at ZT6 in *Bdr* mutants versus wild-type controls (n = 4–5; ZT6: p = 0.0198, ANOVA, planned comparison). (B and C) Representative ISH of the mid-SCN from *Bdr* and control mice using *Avp* and *Vip* riboprobes (B). The significantly advanced peak of expression of *Avp* at ZT6 in mutants was confirmed by quantification of the ISH signal (n = 3; ZT6: p = 0.003, ANOVA) (C). (D and E) Immunohistochemistry of Avp over 24 hr confirmed that Avp expression peaked at ZT10 in wild-type SCN (D) but ZT6 in *Bdr* mutants, as quantified (E) (n = 4; p values: ZT6 = 0.001, ZT10 = 0.002, ZT22 = 0.015, ANOVA). (F) The circadian timing of serum corticosterone levels were also significantly advanced in *Bdr* mice compared to wild-type (n = 9; p values: ZT2 = 0.013, ZT6 = 0.018, ZT10 = 0.016, ZT14 = 0.037, ZT18 = 0.008, ANOVA). Data are presented as mean ± SEM.

To validate the microarray findings, we undertook qPCR and confirmed that *Avp* showed a significantly elevated level of gene expression at ZT6 versus ZT11 in *Bdr* mice when compared to wild-type controls (Figure 4A); these findings were also replicated by in situ hybridization (ISH) (Figures 4B and 4C). Upregulation of *Nts* from ZT6 to ZT11 in the *Bdr* SCN was also confirmed by qRT-PCR (Figure S4E), but changes in *Tac1* fell below significance (Figure S4F). Altered phasing of Avp protein was then demonstrated by immunohistochemistry in an independent cohort of animals sampled over 24 hr. The Avp peak within the SCN of *Bdr* mutants was at ZT6, whereas in wild-type mice, expression peaked at ZT10 (Figures 4D and 4E). Microarray analysis and ISH profiling of the SCN output neuropeptide vasoactive intestinal peptide (*Vip*) were comparable between genotypes, however (Figure 4B). *Per2:Luc* reporter results were also validated by microarray analysis and qRT-PCR of the molecular clock genes *Bmal1*, *Clock*, *Per1*, and *Per2*. None showed any significant differences between mutant and wild-type mice (Figures S4B–S4D).

The circadian control of glucocorticoid production is strongly dependent upon the SCN [24]. As a consequence, we measured plasma corticosterone levels as an additional assay, along with locomotor behavior, of a peripheral rhythm under circadian control. Serum corticosterone measurements were taken across the 24 hr circadian day and were found to be significantly advanced in the *Bdr* mice when compared to wild-type controls (Figure 4F), correlating with the *Bdr* phase advance observed in both SCN Avp expression and wheel-running activity rhythms.

## Discussion

Although no definitive causal links between SNAP-25 mutations and schizophrenia have been proven, there are a considerable number of studies that demonstrate an association between this gene and mental health. For example, synaptic dysfunction and abnormal neurotransmitter release are thought to underpin many neuropsychiatric disorders, including schizophrenia [25, 26]. The *Bdr Snap-25* missense mutation results in increased binding affinities within the soluble NSF attachment protein receptor (SNARE) complex, leading to impaired exocytotic vesicle recycling and exocytosis and a reduction in the amplitude of excitatory postsynaptic potentials [3]. Importantly, we have shown previously that combining the *Bdr* mutation with a prenatal environmental insult produces enhanced behavioral endophenotypes; the sensorimotor gating (prepulse inhibition) deficit observed in mutants was not only exacerbated by prenatal stress but was also reversible with antipsychotic treatment [4]. Notably, recent data has shown *Bdr*-like enhanced SNAP-25 binding at the striatal synapse in schizophrenia patients [16]. There is also some indirect evidence that Snap-25 may play a role in the circadian system; the gene has a rhythmic 24 hr pattern of expression in the rodent SCN [27], and the effects of Botulinium toxin A administration to the SCN in vitro suggests an important general role for vesicle cycling in clock cell regulation [28]. Finally, there are additional links between SNAP-25 and human cognitive endophenotypes [29] and multiple reports of altered SNAP-25 expression in both patients [12, 30] and mouse models of schizophrenia [31]. Collectively, these findings support the case that the *Bdr* mutant provides a powerful model to study the relationship between circadian disturbance and synaptic abnormalities in neuropsychiatric disease.

The results in this study show that the rest and activity cycle of *Bdr* mice is phase advanced and markedly fragmented under a 12:12 LD cycle. However, there appear to be no defects in the retinal input to the circadian system or in the capacity of the SCN of the *Bdr* mouse to generate a normal circadian rhythm under constant conditions. By contrast, the 24 hr rhythms of both Avp, the best characterized output peptide of the SCN, and plasma coticosterone are significantly phased advanced. Collectively, these results suggest that Snap-25 plays a critical role in the synchronization of central and peripheral circadian oscillators, coupling molecular rhythms within the SCN to key output signals that in turn drive locomotor rhythms of behavior and corticosterone [23]. In the case of Avp, the fact that both mRNA and protein are phase advanced suggests that the synaptic defects caused by mutant Snap-25 must be afferent to the Avp neurons in the SCN (see graphical abstract for summary). We have shown previously that the *Bdr* mutation results in attenuated neurotransmitter release under sustained stimulation [3]. It is possible, therefore, that the SCN drive on output pathways under LD conditions diminishes over time, and this may account for the earlier phase of output signals from the SCN.

Internal desynchrony and circadian misalignment are seen in many neuropsychiatric diseases and are thought to involve dysfunction of neurotransmitter systems [1, 32]. Schizophrenia is associated with significant circadian disruption, the core aspects of which include abnormal phasing, rest and activity fragmentation, and reduced stability in rest and activity behavior, as illustrated here by a typical human activity profile (Figure S2) [1, 17–19]. A recent study has provided the most comprehensive analysis to date on sleep in schizophrenia, demonstrating that rest and activity disturbance is not an artifact of antipsychotic treatment or lack of employment [20]. Although direct comparisons between human and rodent circadian behaviors can be problematic, many aspects of the circadian disturbance seen in *Bdr* mice are similar to that described in patients. In addition, we observed considerable heterogeneity in the rest and activity phenotype in *Bdr* mutants ranging from robust and entrained rhythms to highly irregular circadian profiles. Such interindividual variation is also seen in patients with schizophrenia and is normally ascribed to the genetic variability typical of any human population [1]. That such wide variation is also found in the circadian phenotype of genetically similar mice, within a comparable environment, is surprising; this suggests that a predisposing gene for a neuropsychiatric disorder could produce either heterogeneity alone or be markedly altered by very subtle environmental factors.

Another notable similarity between schizophrenia and our findings in *Bdr* mice is perturbation of the hypothalamo-pituitary-adrenal (HPA) axis [33]. Such disruption would not feed back directly onto the SCN, however, because the SCN lacks glucocorticoid receptors and is resistant to such peripheral rhythm disturbance [34]. Interestingly, abnormal phasing of glucocorticoids has been reported in patients with schizophrenia [35]. Furthermore, a rodent model of Avp disruption displays schizophrenic endophenotypes [36], and levels of both AVP and NTS have been reported to be altered in schizophrenia [37, 38]. Significantly, both of these neuropeptides are currently under investigation as potential antipsychotic therapeutic targets [39, 40].

In summary, the *Bdr* model of schizophrenia-associated synaptic dysfunction provides the first tangible link between disruption of rest and activity cycles and the mechanisms underlying neuropsychiatric disorders. Our results suggest that the *Bdr* rest and activity phenotype arises from a disruption of synaptic connectivity that causes desynchrony between the SCN and peripheral rhythms. These findings argue strongly that sleep disruption in schizophrenia can occur independently of pharmaceutical or environmental cues. Thus an abnormality in neurotransmitter signaling that predisposes an individual to neuropsychiatric illness can have a direct impact upon sleep/wake timing. As a result, circadian disruption may serve as a useful endophenotype to assess familial predisposition to schizophrenia. Furthermore, because of this mechanistic overlap, it is possible that many of the comorbid pathologies found in mental health arise directly from or are exacerbated by disrupted sleep. The stabilization of sleep in such individuals suggests a means to reduce symptoms and improve quality of life.

## Experimental Procedures

See Supplemental Experimental Procedures for details.

## Accession Numbers

Data from the microarray expression analysis are available online under ArrayExpress accession number E-MEXP-3493.

## Supplemental Information

Document S1. Figures S1–S4, Tables S1–S4, and Supplemental Experimental Procedures

## References

[1] Wulff K., Porcheret K., Cussans E., Foster R.G. (2009). Sleep and circadian rhythm disturbances: multiple genes and multiple phenotypes. Curr. Opin. Genet. Dev..

[2] Ruhrmann S., Schultze-Lutter F., Salokangas R.K., Heinimaa M., Linszen D., Dingemans P., Birchwood M., Patterson P., Juckel G., Heinz A. (2010). Prediction of psychosis in adolescents and young adults at high risk: results from the prospective European prediction of psychosis study. Arch. Gen. Psychiatry.

[3] Jeans A.F., Oliver P.L., Johnson R., Capogna M., Vikman J., Molnár Z., Babbs A., Partridge C.J., Salehi A., Bengtsson M. (2007). A dominant mutation in Snap25 causes impaired vesicle trafficking, sensorimotor gating, and ataxia in the blind-drunk mouse. Proc. Natl. Acad. Sci. USA.

[4] Oliver P.L., Davies K.E. (2009). Interaction between environmental and genetic factors modulates schizophrenic endophenotypes in the Snap-25 mouse mutant blind-drunk. Hum. Mol. Genet..

[5] Carroll L.S., Kendall K., O'Donovan M.C., Owen M.J., Williams N.M. (2009). Evidence that putative ADHD low risk alleles at SNAP25 may increase the risk of schizophrenia. Am. J. Med. Genet. B. Neuropsychiatr. Genet..

[6] Fanous A.H., Zhao Z., van den Oord E.J., Maher B.S., Thiselton D.L., Bergen S.E., Wormley B., Bigdeli T., Amdur R.L., O'Neill F.A. (2010). Association study of SNAP25 and schizophrenia in Irish family and case-control samples. Am. J. Med. Genet. B. Neuropsychiatr. Genet..

[7] Lewis C.M., Levinson D.F., Wise L.H., DeLisi L.E., Straub R.E., Hovatta I., Williams N.M., Schwab S.G., Pulver A.E., Faraone S.V. (2003). Genome scan meta-analysis of schizophrenia and bipolar disorder, part II: Schizophrenia. Am. J. Hum. Genet..

[8] Arinami T., Ohtsuki T., Ishiguro H., Ujike H., Tanaka Y., Morita Y., Mineta M., Takeichi M., Yamada S., Imamura A., Japanese Schizophrenia Sib-Pair Linkage Group (2005). Genomewide high-density SNP linkage analysis of 236 Japanese families supports the existence of schizophrenia susceptibility loci on chromosomes 1p, 14q, and 20p. Am. J. Hum. Genet..

[9] Thompson P.M., Egbufoama S., Vawter M.P. (2003). SNAP-25 reduction in the hippocampus of patients with schizophrenia. Prog. Neuropsychopharmacol. Biol. Psychiatry.

[10] Thompson P.M., Kelley M., Yao J., Tsai G., van Kammen D.P. (2003). Elevated cerebrospinal fluid SNAP-25 in schizophrenia. Biol. Psychiatry.

[11] Fatemi S.H., Earle J.A., Stary J.M., Lee S., Sedgewick J. (2001). Altered levels of the synaptosomal associated protein SNAP-25 in hippocampus of subjects with mood disorders and schizophrenia. Neuroreport.

[12] Johnson R.D., Oliver P.L., Davies K.E. (2008). SNARE proteins and schizophrenia: linking synaptic and neurodevelopmental hypotheses. Acta Biochim. Pol..

[13] Spellmann I., Müller N., Musil R., Zill P., Douhet A., Dehning S., Cerovecki A., Bondy B., Möller H.J., Riedel M. (2008). Associations of SNAP-25 polymorphisms with cognitive dysfunctions in Caucasian patients with schizophrenia during a brief trail of treatment with atypical antipsychotics. Eur. Arch. Psychiatry Clin. Neurosci..

[14] Young C.E., Arima K., Xie J., Hu L., Beach T.G., Falkai P., Honer W.G. (1998). SNAP-25 deficit and hippocampal connectivity in schizophrenia. Cereb. Cortex.

[15] Müller D.J., Klempan T.A., De Luca V., Sicard T., Volavka J., Czobor P., Sheitman B.B., Lindenmayer J.P., Citrome L., McEvoy J.P. (2005). The SNAP-25 gene may be associated with clinical response and weight gain in antipsychotic treatment of schizophrenia. Neurosci. Lett..

[16] Barakauskas V.E., Beasley C.L., Barr A.M., Ypsilanti A.R., Li H.Y., Thornton A.E., Wong H., Rosokilja G., Mann J.J., Mancevski B. (2010). A novel mechanism and treatment target for presynaptic abnormalities in specific striatal regions in schizophrenia. Neuropsychopharmacology.

[17] Wulff K., Joyce E., Middleton B., Dijk D.J., Foster R.G. (2006). The suitability of actigraphy, diary data, and urinary melatonin profiles for quantitative assessment of sleep disturbances in schizophrenia: a case report. Chronobiol. Int..

[18] Martin J., Jeste D.V., Caliguiri M.P., Patterson T., Heaton R., Ancoli-Israel S. (2001). Actigraphic estimates of circadian rhythms and sleep/wake in older schizophrenia patients. Schizophr. Res..

[19] Martin J.L., Jeste D.V., Ancoli-Israel S. (2005). Older schizophrenia patients have more disrupted sleep and circadian rhythms than age-matched comparison subjects. J. Psychiatr. Res..

[20] Wulff K., Dijk D.J., Middleton B., Foster R.G., Joyce E. (2011). Sleep and circadian dysruption in schizophrenia patients. Br. J. Psychiatry.

[21] Fisher S.P., Godinho S.I., Pothecary C.A., Hankins M.W., Foster R.G., Peirson S.N. (2011). Rapid assessment of sleep/wake behaviour in mice. J. Biol. Rhythms.

[22] Reghunandanan V., Reghunandanan R. (2006). Neurotransmitters of the suprachiasmatic nuclei. J. Circadian Rhythms.

[23] Colwell C.S. (2011). Linking neural activity and molecular oscillations in the SCN. Nat. Rev. Neurosci..

[24] Dickmeis T. (2009). Glucocorticoids and the circadian clock. J. Endocrinol..

[25] Waites C.L., Garner C.C. (2011). Presynaptic function in health and disease. Trends Neurosci..

[26] Stephan K.E., Baldeweg T., Friston K.J. (2006). Synaptic plasticity and dysconnection in schizophrenia. Biol. Psychiatry.

[27] Panda S., Antoch M.P., Miller B.H., Su A.I., Schook A.B., Straume M., Schultz P.G., Kay S.A., Takahashi J.S., Hogenesch J.B. (2002). Coordinated transcription of key pathways in the mouse by the circadian clock. Cell.

[28] Deery M.J., Maywood E.S., Chesham J.E., Sládek M., Karp N.A., Green E.W., Charles P.D., Reddy A.B., Kyriacou C.P., Lilley K.S., Hastings M.H. (2009). Proteomic analysis reveals the role of synaptic vesicle cycling in sustaining the suprachiasmatic circadian clock. Curr. Biol..

[29] Golimbet V.E., Alfimova M.V., Gritsenko I.K., Lezheiko T.V., Lavrushina O.M., Abramova L.I., Kaleda V.G., Barkhatova A.N., Sokolov A.V., Ebstein R.P. (2010). Association between a synaptosomal protein (SNAP-25) gene polymorphism and verbal memory and attention in patients with endogenous psychoses and mentally healthy subjects. Neurosci. Behav. Physiol..

[30] Corradini I., Verderio C., Sala M., Wilson M.C., Matteoli M. (2009). SNAP-25 in neuropsychiatric disorders. Ann. N Y Acad. Sci..

[31] Pletnikov M.V., Ayhan Y., Nikolskaia O., Xu Y., Ovanesov M.V., Huang H., Mori S., Moran T.H., Ross C.A. (2008). Inducible expression of mutant human Dros. Inf. Serv.C1 in mice is associated with brain and behavioral abnormalities reminiscent of schizophrenia. Mol Psychiatry.

[32] Lisman J.E., Coyle J.T., Green R.W., Javitt D.C., Benes F.M., Heckers S., Grace A.A. (2008). Circuit-based framework for understanding neurotransmitter and risk gene interactions in schizophrenia. Trends Neurosci..

[33] Bradley A.J., Dinan T.G. (2010). A systematic review of hypothalamic-pituitary-adrenal axis function in schizophrenia: implications for mortality. J. Psychopharmacol. (Oxford).

[34] Balsalobre A., Brown S.A., Marcacci L., Tronche F., Kellendonk C., Reichardt H.M., Schütz G., Schibler U. (2000). Resetting of circadian time in peripheral tissues by glucocorticoid signaling. Science.

[35] Hempel R.J., Tulen J.H., van Beveren N.J., Röder C.H., de Jong F.H., Hengeveld M.W. (2010). Diurnal cortisol patterns of young male patients with schizophrenia. Psychiatry Clin. Neurosci..

[36] Feifel D., Shilling P.D., Melendez G. (2011). Further characterization of the predictive validity of the Brattleboro rat model for antipsychotic efficacy. J. Psychopharmacol. (Oxford).

[37] Cáceda R., Kinkead B., Nemeroff C.B. (2006). Neurotensin: role in psychiatric and neurological diseases. Peptides.

[38] De Wied D., Sigling H.O. (2002). Neuropeptides involved in the pathophysiology of schizophrenia and major depression. Neurotox. Res..

[39] Meyer-Lindenberg A., Domes G., Kirsch P., Heinrichs M. (2011). Oxytocin and vasopressin in the human brain: social neuropeptides for translational medicine. Nat. Rev. Neurosci..

[40] Kinkead B., Nemeroff C.B. (2006). Novel treatments of schizophrenia: targeting the neurotensin system. CNS Neurol. Disord. Drug Targets.

