# Supplementary material for: Disrupted Circadian Rhythms in a Mouse Model of Schizophrenia
Source: Curr Biol. 2012 Feb 21;22(4):314–9. doi: 10.1016/j.cub.2011.12.051 (PMC3356578; doi:10.1016/j.cub.2011.12.051)
Supplement: Document S1. Figures S1–S4, Tables S1–S4, and Supplemental Experimental Procedures [file mmc1.pdf]

Current Biology, Volume *22*

Supplemental Information

Disrupted Circadian Rhythms

in a Mouse Model Of Schizophrenia

Peter L. Oliver, Melanie V. Sobczyk, Elizabeth S. Maywood, Benjamin Edwards, Sheena Lee, Achilleas Livieratos, Henrik Oster, Rachel Butler, Sofia I.H. Godinho, Katharina Wulff, Stuart N. Peirson, Simon P. Fisher, Johanna E. Chesham, Janice W. Smith, Michael H. Hastings, Kay E. Davies, and Russell G. Foster

Supplemental Inventory

1. Supplemental Figures and Tables

Figure S1, related to Figures 1–3

Figure S2, related to Figure 1

Figure S3, related to Figure 3

Figure S4, related to Figure 4

Table S1, related to Figures 1–3

Table S2, related to Figures 1–3

Table S3, related to Figure 4

Table S4, related to Figure 4

2. Supplemental Experimental Procedures

3. Supplemental References

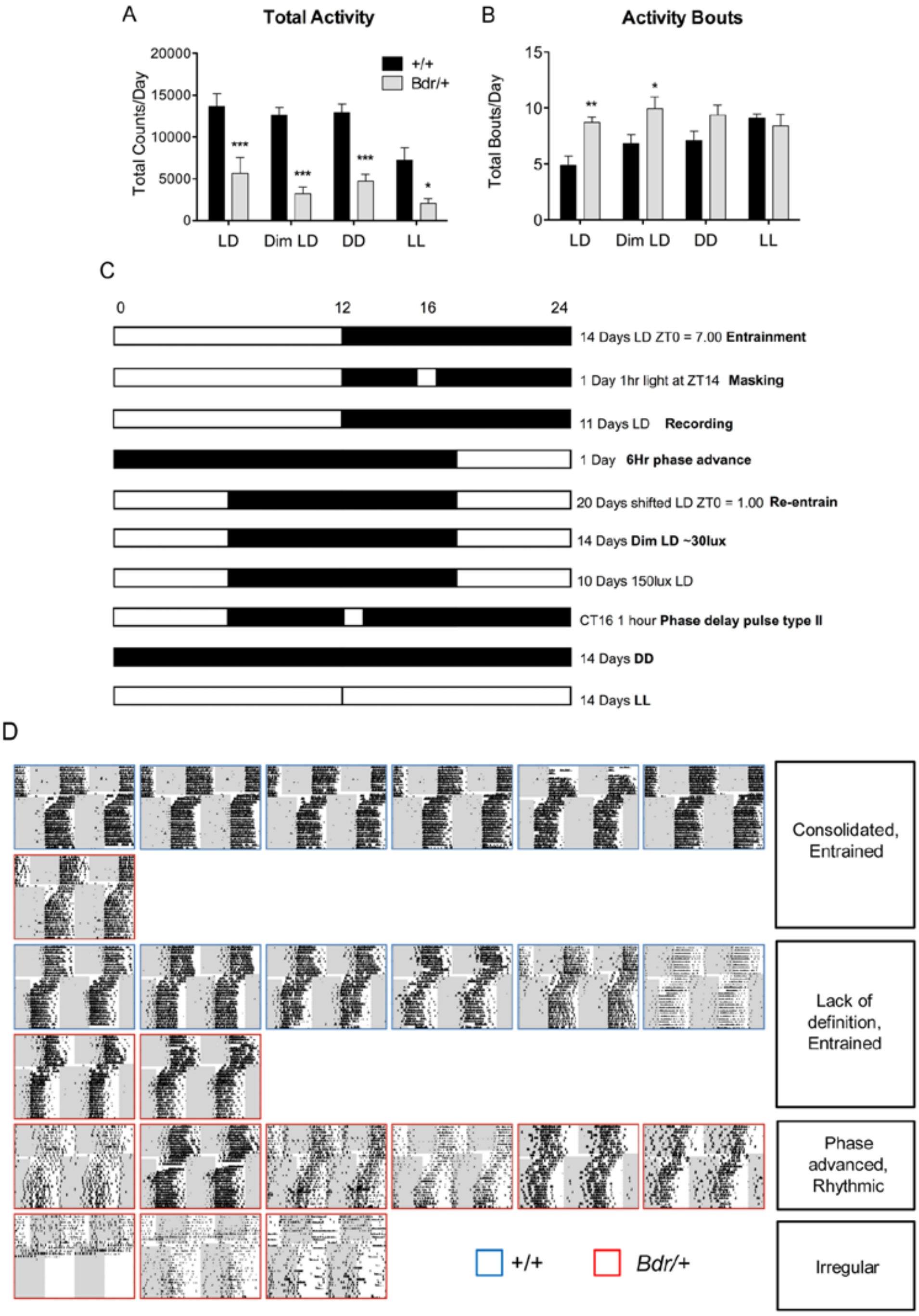
A
Total Activity
Total Counts/Day
20000
15000
10000
5000
0
+/+
Bdr/+
***
***
***
*
LD
Dim LD
DD
LL
B
Activity Bouts
Total Bouts/Day
15
10
5
0
**
*
LD
Dim LD
DD
LL
C
0
12
16
24
14 Days LD ZT0 = 7.00 Entrainment
1 Day 1hr light at ZT14 Masking
11 Days LD Recording
1 Day 6Hr phase advance
20 Days shifted LD ZT0 = 1.00 Re-entrain
14 Days Dim LD ~30lux
10 Days 150lux LD
CT16 1 hour Phase delay pulse type II
14 Days DD
14 Days LL
D
Consolidated, Entrained
Lack of definition, Entrained
Phase advanced, Rhythmic
Irregular
+/+
Bdr/+

Figure S1. *Bdr* Wheel-Running Activity Is Reduced and Subject to Increased Bouts / Summary of Circadian Screen / Heterogeneity of Wheel Running Data, Related to Figures 1–3

(A and B) Overall *Bdr* wheel-running activity is significantly reduced across all light conditions (A) and there is a general increase of activity bouts in the *Bdr* animals which is significant under LD relative to wild-type controls (B). See table S2 for related *p*-values.

(C) The circadian wheel-running screen. The numbers along the top denote circadian timing under the initial 12:12 light/dark (LD) cycle. Zeitgeber time (ZT) of all environmental lighting changes is stated in the brief description of a given paradigm down the right hand side of the figure. The black (dark) and white (light) bars graphically represent the changing light environment as the screen progresses. All lights are set at an intensity of 150 lux unless otherwise stated. Further abbreviations: DD – constant dark, CT – Circadian time and LL - constant light.

(D) *Bdr* and wild-type circadian phenotypic heterogeneity under 150 lux LD and a 6-hr phase advance. Actograms from all 24 animals that were subjected to the behavioural screen in under a 12:12 hr LD cycle (11 days) at 150 lux and a 6 hr phase advance (18 days) are shown. Wild-type actograms (blue border) clearly all show entrainment to the light cycle and relatively low light phase activity or rhythmic fragmentation*. Bdr* actograms (red border) span from the robustly rhythmic and entrained to the highly irregular. Early termination of the screen due to illness resulted in one shortened actogram. Assessment of phenotypic distribution using a chi-squared test (robustly entrained vs. abnormal) indicates that the *Bdr* animals are significantly more disrupted than the wild-type controls (χ^2 = 0.0247). Prior to the behavioural screen, experimental animals were weighed as neonates, at weaning and prior to testing to confirm that no overt developmental delay could account for the phenotypic heterogeneity (data not shown).

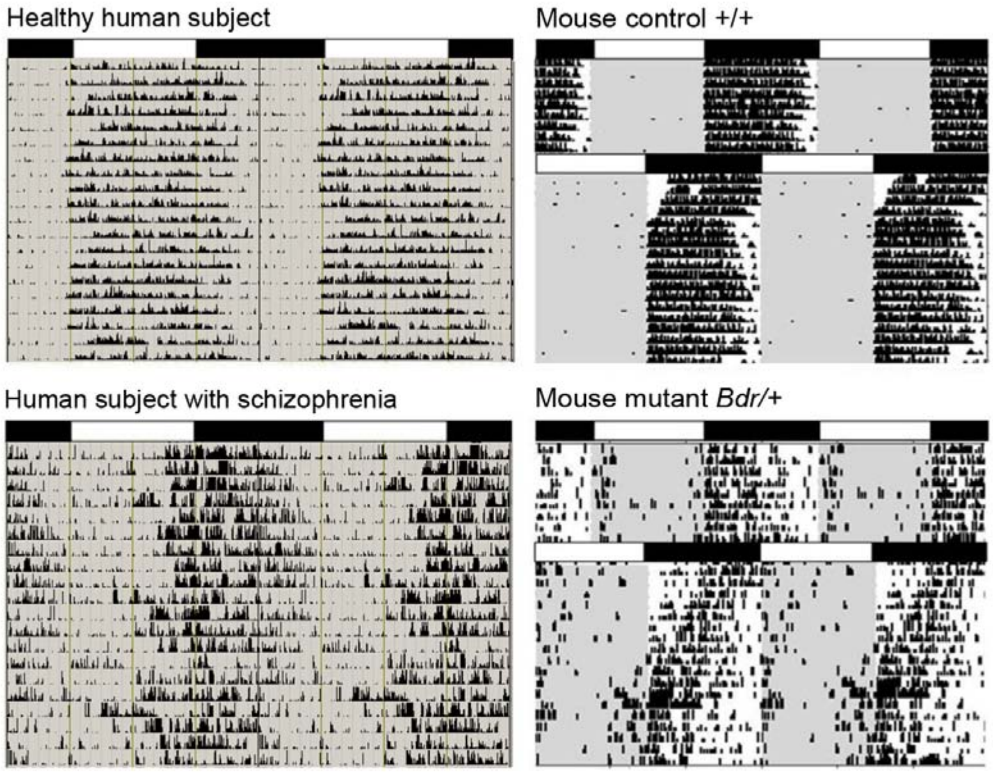

Figure S2. Comparison of Human and Mouse Activity Profiles, Related to Figure 1

The rest/activity profiles of a human subject with schizophrenia illustrates that circadian reversal and activity fragmentation, often accredited to schizophrenia, is also observed in *Bdr* mice compared to the more stable profile of controls in each case. For details of the human subjects, see Supplemental Experimental Procedures.

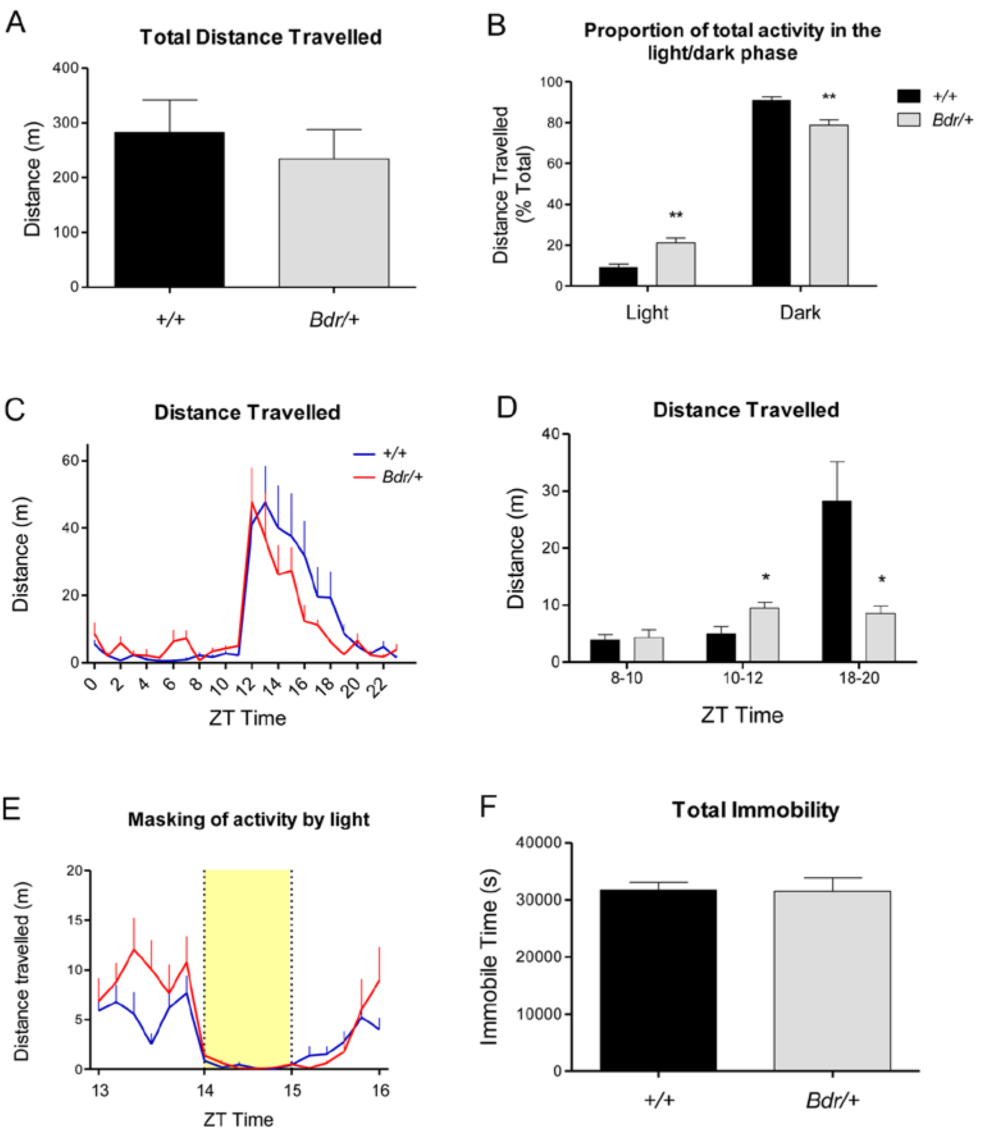

Figure S3. Video Tracking Data, Related to Figure 3

Bdr and wild-type mice were observed over a 24-hr 12:12 LD cycle (N = 6). There was no significant difference in the total distance travelled (p = 0.5648, t-test) (A), although *Bdr* mice travelled proportionally more in the light phase and less in the dark phase (2-way ANOVA main effect of genotype: p = 0.0033) (B). This phase advance of activity is also reflected in the total distance travelled plotted over 24 hr (C) and quantified by a significant increase in the distance travelled by *Bdr* mice compared to controls in the last 2 hr of the light phase (ZT10-12) compared with a significant decrease towards the end of the dark phase (ZT18-20) (2-way ANOVA effect of genotype: p = 0.0011; Bonferroni post-hoc tests: ZT8-10 p = 0.8067 , ZT10-12 p = 0.0249 , ZT18-20 p = 0.0321) (D). Mice of both genotypes showed a robust masking of activity by light at ZT14 to ZT15 (2-way ANOVA main effect of genotype: p = 0.976) (E). As a correlate of sleep, there was no difference in total immobility over 24 hr between genotypes (p = 0.9284, t-test) (F). All data are expressed as mean ± SEM. Assessment of immobile episodes and therefore sleep fragmentation in the light phase did not show any significant differences between genotypes (data not shown).

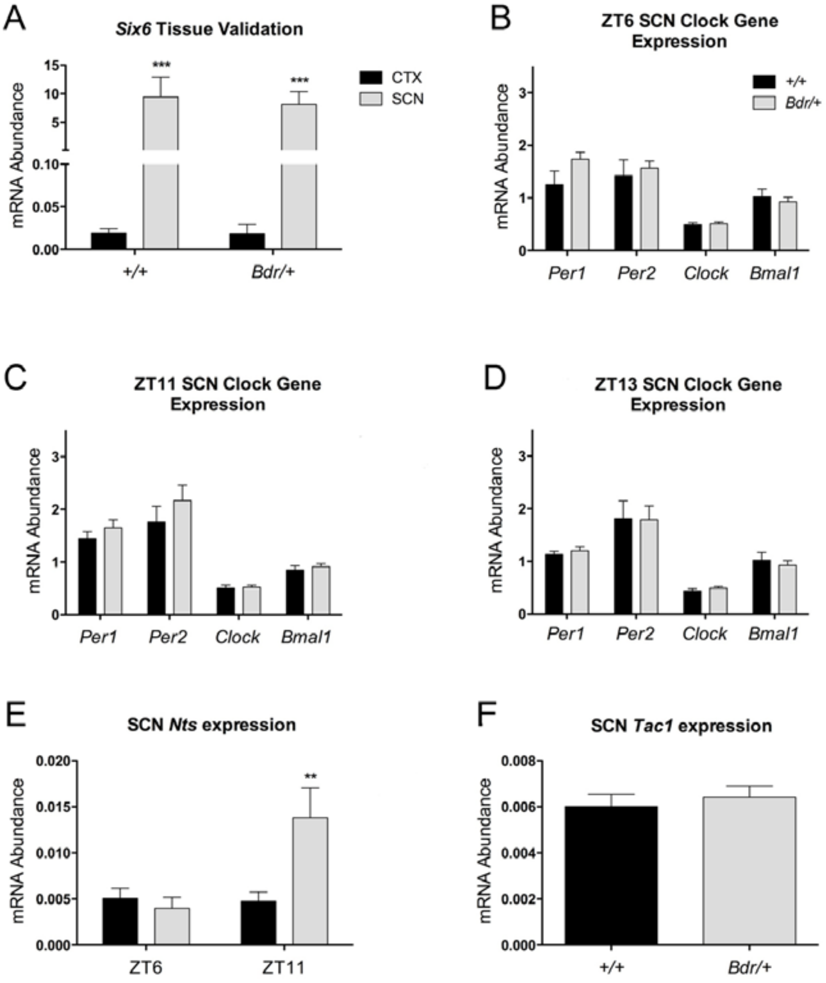

Figure S4. SCN Tissue and Gene Expression Validation Using qPCR, Related to Figure 4

(A) The relative abundance of *Six6* mRNA in the SCN compared to the cortex (CTX) is shown. There was no significant effect of genotype and *Six6* expression in the SCN (N = 12-15) for wild-type (p = *0.00034,* ANOVA, planned comparison) and *Bdr* (p = 0.00023, ANOVA) cohorts as shown.

(B-D) SCN clock gene expression at ZT6 (B), ZT11 (C) and ZT13 (D) show comparable mRNA abundance between *Bdr* and wild-type cohorts; there was no effect of genotype on SCN clock gene expression at any zeitgeber time (ZT) (2-way ANOVA main effect of genotype: p = 0.2647).

(E) *Nts* (Neurotensin) expression is significantly increased at ZT11 in *Bdr* mice (N = 4, p = 0.02864*,* ANOVA) compared to the wild-types with comparable levels at ZT11.

(F) *Bdr* mice show comparable *Tac1* (substance P) expression to wild-type controls at both ZT6 and ZT11. All data expressed as mean ± SEM.

Table S1. Selected Parameters Used for Analysis of Circadian Screening in the *Bdr* Cohort, Related to Figures 1–3

**Parameter**	**Units**	**Definition**
Mean Activity Onset	Hrs<br>(Clock time)	The average clock time of activity onset, this is a robust indicator of entrainment to light under a given LD cycle and can be interpreted as the phase angle of entrainment (Ψ) if subtracted from the clock value for ZT12 (lights off)
Periodogram Tau	Hrs	Calculation of the circadian period using the Chi-squared periodogram method (Sokolove and Bushell, 1978)
Periodogram Amplitude	AU	Indicator of rhythm stability and consistency, the zenith of a periodogram curve.
Total Activity	Total Counts	Total average counts over the 24 hr day
Relative Light Activity	%	Total average counts over the 12 hr light period expressed as a percentage of total activity
Total Bouts	Bouts/day	A bout of activity is defined by a period of activity ≥10 min which is preceded or followed by a rest period of ≥10 min
Relative Masking Activity	%	The total masking pulse activity is expressed as a percentage of the pre-pulse masking activity to normalise for activity level differences between individual animals
Phase Advance Activity Onsets	Hrs<br>(Clock time)	The activity onsets are plotted against the days elapsed from previous to, during and after the phase advance to represent the re-entrainment of the clock to the new light cycle
Type II Phase Delay Phase Shift	Hrs	Regression lines are fitted to the onsets preceding and following the phase shift paradigm. The difference between these regression lines on the day of the phase shift pulse is expressed as the phase shift in hr +/- from the original phase of entrainment

Table S2. Circadian Screen Descriptive Statistics and p Values, Related to Figures 1-3

**Parameter**	**Condition**	**+/+ (mean ± SEM)**	***Bdr*/+ (mean ± SEM)**	**p value**
Total activity/day	LD	13640 ± 1554 N=12	5645 ± 1913 N=10	0.0373
Total activity/day	Dim LD	12630 ± 881.9 N=9	3231 ± 813.7 N=9	0.0000
Total activity/day	DD	12930 ± 1036 N=9	4711 ± 832.4 N=9	0.0000
Total activity/day	LL	7246 ± 1473 N=9	2077 ± 532.0 N=8	0.0067
Phase of entrainment	LD	0.051 ± 0.027 N=12	-0.308 ± 0.132 N=10	0.0269
Phase of entrainment	Dim LD	-0.760 ± 0.280 N=9	-2.183 ± 0.866 N=9	0.1374
% Light activity	LD	9.775 ± 1.251 N=12	28.517 ± 6.106 N=10	0.0132
% Light activity	Dim LD	11.788 ± 1.633 N=9	33.391 ± 8.404 N=9	0.0226
Total bouts/day	LD	3.088 ± 0.333 N=12	6.435 ± 0.666 N=10	0.0127
Total bouts/day	Dim LD	68.00 ± 8.042 N=9	99.33 ± 10.77 N=9	0.0612
Total bouts/day	DD	7.104 ± 0.840 N=9	9.383 ± 0.893 N=9	0.1115
Total bouts/day	LL	9.089 ± 0.393 N=9	8.388 ± 1.060 N=8	0.5256
P.Gram amplitude	LD	835.0 ± 48.79 N=12	471.6 ± 61.84 N=10	0.0027
P.Gram amplitude	Dim LD	728.7 ± 47.47 N=9	416.8 ± 41.68 N=10	0.0004
P.Gram amplitude	DD	852.1 ± 63.59 N=9	523.8 ± 69.98 N=9	0.0061
P.Gram amplitude	LL	353.1 ± 15.59 N=9	283.9 ± 16.06 N=8	0.0075
P.Gram Tau	DD	23.52 ± 0.098 N=9	23.50 ± 0.108 N=9	0.8823
P.Gram Tau	LL	25.61 ± 0.277 N=9	25.79 ± 0.497 N=8	0.7469
Type II Phase Delay	N/A	1.646 ± 0.210 N=10	1.749 ± 0.168 N=9	0.7116
Negative Masking	10 lux LP	29.61 ± 5.382 N=5	30.89 ± 6.166 N=4	0.8801

Negative Masking	150 lux LP	2.691 ± 1.552 N=6	1.218 ± 1.150 N=4	0.4031
Interdaily Stability	LD	1.771 ± 0.2327 N=12	0.7704 ± 0.1109 N=11	0.0011
Intradaily variability	LD	0.9103 ± 0.08986 N=12	1.410 ± 0.1428 N=11	0.0065

The above table displays all graphically represented values and related planned comparison *p*-values within a multivariate one-way ANOVA to assess the effect of genotype across individual lighting condition(s) (150 lux 12:12 LD (LD), 30 lux 12:12 LD (Dim LD), constant darkness (DD), 150 lux constant light (LL), and light pulse (LP)) for each measurement, except for in the case of type II phase delay, interdaily stability and intradaily variability analysis where an unpaired Student's *t*-tests were used. Significant main effects of genotype were observed in measures of total activity (p = 0.0008, ANOVA), % light activity (p = 0.0314, ANOVA), total bouts (p = 0.03816, ANOVA) and periodogram amplitude (p = 0.0118, ANOVA). P.Gram refers to periodogram.

Table S3. Comparison of Wild-Type (WT) and *Bdr* SCN Transcriptional Profiles at ZT6, 11 and 13, Related to Figure 4

Gene ID	Gene name	Affymetrix transcript ID	Fold change
**ZT6 *Bdr* vs WT**			
Tac1	tachykinin 1	10536363	1.9
Lbp	lipopolysaccharide binding protein	10478048	-1.7
C4a/b	complement component 4A/B	10450242	1.7
Ptpn	protein tyrosine phosphatase, non-receptor type 3	10513158	1.6
Rassf9	Ras association (RalGDS/AF-6) domain family (N-terminal) member 9	10366153	-1.5
Efmp1	epidermal growth factor-containing fibulin-like extracellular matrix protein 1	10374777	-1.5
**ZT11 *Bdr* vs WT**			
Dcn	Decorin	10365974	-1.5
C4a/b	complement component 4A/B	10450242	1.5
**ZT13 *Bdr* vs WT**			
SNORD104	-	10382104	-1.5
Gfap	glial fibrillary acidic protein	10391798	1.5

All statistically significant differences over 1.5-fold are shown. Down-regulation of a transcript in *Bdr* versus WT is indicated using a negative symbol.

Table S4. Temporal Analysis of SCN Microarray Expression Data, Related to Figure 4

Gene ID	Gene name	Affymetrix Transcript ID	Fold change
**ZT6 vs ZT13 unique to *Bdr***			
Avp	arginine vasopressin	10487685	-3.3
Nts	Neurotensin	10372139	3.1
Tac1	tachykinin 1	10536363	-2.0
Rasl11b	RAS-like, family 11, member B	10522467	-2.0
Rgs16	regulator of G-protein signaling 16	10350733	-2.0
Brs3	bombesin-like receptor 3	10599776	2.0
Rnu2	U2 small nuclear RNA	10578688	-2.0
Rnu2	U2 small nuclear RNA	10381458	-1.9
Rnu2	U2 small nuclear RNA	10391488	-1.9
Rnu2	U2 small nuclear RNA	10381460	-1.9
Rnu2	U2 small nuclear RNA	10381470	-1.9
Rnu2	U2 small nuclear RNA	10381472	-1.9
---	SNORD60	10442493	-1.8
Rnu2	U2 small nuclear RNA	10603736	-1.8
U1	U1 snRNA	10587778	-1.8
Fezf1	Fez family zinc finger 1	10543362	-1.8
Dcn	Decorin	10365974	1.7
Efemp1	epidermal growth factor-containing fibulin-like extracellular matrix protein 1	10374777	1.7
D330045A20Rik	RIKEN cDNA D330045A20 gene	10601993	1.7
Gm5502	predicted gene 5502	10457927	-1.7
Snord33	small nucleolar RNA, C/D box 33	10563112	-1.7
Glra1	glycine receptor, alpha 1 subunit	10386076	-1.6
Foxg1	forkhead box G1	10395596	1.6
Ecel1	endothelin converting enzyme-like 1	10356379	1.6
Mc4r	melanocortin 4 receptor	10459512	1.6
ND6	NADH dehydrogenase subunit 6	10598087	-1.6
4930467E23Rik	RIKEN cDNA 4930467E23	10570634	-1.6
Clic6	chloride intracellular channel 6	10436958	1.6
C1ql1	complement component 1, q subcomponent-like 1	10391828	1.5
Hpcal1	hippocalcin-like 1	10394778	1.5
Prlr	prolactin receptor	10423030	1.5
Dgkk	diacylglycerol kinase kappa	10598251	1.5
**ZT6 vs ZT11 unique to *Bdr***			
Nts	Neurotensin	10372139	3.8
Brs3	bombesin-like receptor 3	10599776	2.0
Rasd1	RAS, dexamethasone-induced 1	10386455	1.8
Prlr	prolactin receptor	10423049	1.7
Gm410	predicted gene 410	10498302	1.6

D19Ertd652e	DNA segment, Chr 19, ERATO Doi 652, expressed	10468415	1.6
Rasl11b	RAS-like, family 11, member B	10522467	-1.6
---	AL713956.13-201	10385234	1.5
Efemp1	epidermal growth factor-containing fibulin-like extracellular matrix protein 1	10374777	1.5

The transcriptional profiles of data from the ZT6 versus ZT13 and ZT6 versus ZT11 timepoints were compared to identify transcripts differentially expressed only in *Bdr* mice. All statistically significant differences over 1.5-fold are shown. Down-regulation of a transcript is indicated using a negative symbol.

Supplemental Experimental Procedures

Animals

Bdr mutants heterozygous for the Snap-25 mutation (*Bdr/*+) [1] were tested with age- and sex-matched littermate wild-type controls (+/+) in all experiments. The *Bdr* mutant line, originated from BALB/c x C3H/HeH mating has since been backcrossed over 20 generations to C3H/HeH. All procedures were conducted in accordance with the United Kingdom Home Office Animals (Scientific Procedures) Act (1986) and approved by the University of Oxford Ethical Review Panel.

Circadian Screening

Age-matched (12 week) male *Bdr* (*Bdr/*+) animals (*N* = 12) with littermate male controls (+/+) were housed in cages (44 x 26 x 12 cm) fitted with running wheels maintained at constant temperature and humidity, with *ad libitum* food and water. 6 cages were arranged in light-controlled chambers with externally controlled fluorescent tubes, supplemented with ND filters to bring the lighting environment down to the required intensity at cage level, as assessed with a cage bottom positioned lux meter. A standardised behavioural screen was developed to examine common circadian paradigms (Figure S1C and Table S1). Data analysis was carried out using 10 min bins of wheel running activity using Clocklab (Actimetrics) and Matlab software. Non-parametric assessments of rhythmicity were carried out using 6 min bins of wheel running activity using Sleep analysis software (Cambridge Neurotechnology Ltd). Negative masking was examined in age-matched (14 week) male *Bdr* and *+/+* littermate control animals (*N* = 6) using a 10 lux and 150 lux one hr light pulse at ZT14. For the type II phase delay, following entrainment to a 150 lux LD cycle, age-matched (12 week) male *Bdr* and *+/+* littermate control animals (*N* = 9-10) were subjected to a day of complete darkness from ZT0 with a 150 lux 1 hr light pulse administered at CT16. This Aschoff type II protocol has been previously used for transgenic mice [2] due to the ease of incorporating it within the circadian screen and to minimise the handling of the animals prior to the light pulse. The phase shift was determined by eye-fitting a line through at least 5 consecutive days of the activity onset in LD preceding the light pulse and in DD following the light pulse [3]. All additional assays were carried out on mice under the same conditions but with no access to running wheels.

Nonparametric Circadian Rhythm Analyses (NPCRA)

The nonparametric analysis of circadian rhythms as originally carried out by Van Someren et al. [4] and was performed using Actiwatch Sleep Analysis software (Cambridge Neurotechnology). Intradaily stability and interdaily variability were assessed using 10 day activity profiles exported from the Actimetrics Clocklab software package in 6 min bins. This method has been previously used to assess circadian rhythmicity in a transgenic mouse model of Huntington's disease [5].

Video Tracking

Age-matched 12 week old male *Bdr* (*N* = 6) and wild-type (*N* = 6) littermate control mice were assessed for activity by video tracking in home cages (44 x 26 x 12 cm) using a method that has been proven to correlate almost identically to EMG / telemetry methods [6]. An infrared-capable camera (Sentient) was fitted above each cage for continuous 24-hr recording within light controlled chambers. Mice had free

access to food and water, but were always in the field of view of the camera. ANY-maze software (Stoelting) recorded activity parameters including distance travelled. Immobile time was defined as a period of immobility of more than 40 seconds and used as a sleep correlate [6]. All mice were entrained to a 12:12 LD (150 lux) cycle for 10 days, followed by a 2 day 12:12 LD (150 lux) habituation period in the cages equipped for video tracking. All mice were subjected to 24 hr of 12:12 LD baseline recording following this 2 day habituation period. The subsequent 24 hr involved a one hr 150 lux light pulse at ZT14 to measure the suppression of activity by light.

Pupillometry

Relative pupil constriction was assessed in 12 week female *Bdr* and wild-type (*N* = 6) mice. Following one hr of dark adaption, the left eye was dilated with atropine and stimulated via a ganzfeld with 480nm (10HBW Andover filtered xenon arc light source) light of 14.6 log quanta. The consensual response of the right eye was recorded using an infrared camera over 27 s. The images were captured at a frame rate of 5-10 fps using a Prosilica gigabit ethernet camera and the data was captured in the form of image files via software provided by BRSL Ltd. running through a CVB platform (Stemmer Imaging). Relative pupil constriction (%) was calculated for each individual mouse by taking the smallest constricted area as a percentage of the pre-constricted maximum pupil size. The pupil area per frame captured was calculated using Image J software (NIH).

Suprachiasmatic Nucleus (SCN) Regional Microarray and qPCR

Age-matched (12 week) male *Bdr* and wild-type animals (*N* = 5) were individually housed and entrained to a 12:12 LD cycle at 150 lux for 10 days prior to the day of tissue collection. Following cervical dislocation, brains were sectioned on a steel matrix between bregma 0 and 1 mm posterior. Tissue punches were taken using a 1 mm sample corer (FST Ltd.) from regions of the SCN and cortex and then snap-frozen. Total RNA was extracted using a phenol chloroform method in combination with the RNeasyMicro kit (Qiagen) and the quality was assessed on a 2100 BioAnalyzer using the RNA 6000 Pico Assay (Agilent Technologies). SYBR green master mix (Invitrogen) qPCR reactions were run on a Step One Real Time PCR System (Applied Biosystems) with two housekeeping genes (*Psmb2* and *Gps1)*. Tissue validation was undertaken using the specific SCN marker *Six6* to assess gene expression levels in the SCN compared to the control cortical sample, normalised to housekeeping gene expression. All Ct values were transformed using 2^{-Ct} prior to target gene expression being normalised to housekeeping gene expression for each independent sample, presented as relative RNA abundance.

For the microarray studies, RNA integrity was first assessed on a BioAnalyzer; all samples had a RNA Integrity Number (RIN) $\geq$ 8 (Agilent Laboratories). Labelled sense ssDNA for hybridization was generated from 110ng starting RNA with the Affymetrix GeneChip WT sense target labeling and control reagents kit (Affymetrix) according to the manufacturer's instructions. Sense ssDNA was fragmented and the distribution of fragment lengths was measured on the BioAnalyser. The fragmented ssDNA was labeled and hybridized to the Affymetrix Mouse Gene 1.0 ST Array (Affymetrix). Chips were processed on an Affymetrix GeneChip Fluidics Station 450 and Scanner 3000. Cel files were generated using Command Console (Affymetrix). Arrays were RMA normalized in GeneSpring GX

11.0 and differentially expressed genes were identified using a Welch *t*-test with a *p*-value cut off of ≤0.05 and a fold change difference of ≥1.5.

In Situ Hybridisation and Light Induction Studies

An independent cohort of age-matched (12 week) male *Bdr* and wild-type animals (*N* = 4) were entrained to a 12:12 LD cycle at 150 lux for 10 days prior to the day of tissue collection at ZT6 and 11. Brains were dissected and snap frozen on dry ice in OCT (VWR), followed by sectioning at 12µM. Regions of *Avp*, *Vip* and *c-fos* (18–502, 290–1318 and 560–1463 bp of GenBank accession numbers NM_009732, NM_011702 and NM_010234 respectively) were used for digoxigenin-labeled riboprobe synthesis and hybridization as described previously [7]. Probes were hybridised for 8 hr for *Avp* and 6 hr for *Vip* and *c-fos*. For the light induction studies, an independent cohort of age-matched (12 week) male *Bdr* and wild-type animals (*N* = 4) were entrained to a 12:12 LD cycle at 150 lux for 10 days. Brains were dissected either before or after a 30 min light pulse at 150 lux at ZT16 on day 11 [8].

Avp Immunoshistochemistry

An independent cohort of age-matched (12 week) male *Bdr* and WT animals (*N* = 4) were entrained to a 12:12 LD cycle at 150 lux for 10 days prior to the day of tissue collection at ZT 2, 6, 10, 14, 18 and 22. Dissected brains were snap frozen on dry ice, followed by sectioning at 12µM. Sections were post-fixed in 4% paraformaldehyde for 4 hr prior to immunostaining. After washes in phosphate buffered saline (PBS), sections were incubated in 3% hydrogen peroxide in methanol and washed again in PBS. Sections were then blocked in 5% normal goat serum with 0.5% Triton X-100, followed by incubation with primary antibodies in the same solution (guinea-pig anti-AVP (Peninsula) 1:5000, 48 hr 4°C). Biotinylated secondary antibody (2 hr, room temperature) and avidin-biotin complex (ABC) (1 hr, room temperature) treatment was carried out using Vectastain ABC method (Vectorlabs). Staining was visualised using diaminobenzidine (DAB) as the substrate for 2 min. Sections were dehydrated and mounted using Histomount (National Diagnostics). For each animal two sections representing the mid-caudal aspect of the SCN were analyzed bilaterally. Sections were analysed using Axiovision 4.6 (Axiovision) and Image J software (NIH). The SCN was measured within a 200 × 400 µm grid and the signal intensity was calculated after correction for the background.

24-Hr Plasma Corticosterone Profile

Age-matched (12 week) male *Bdr* and wild-type animals (*N* = 8) were entrained to a 12:12 LD cycle at 150 lux for 10 days prior blood collection at ZT2, 6, 10, 14, 18 and 22. Plasma corticosterone measurements were calculated using the corticosterone EIA kit (Immunodiagnostic Systems) according to the manufacturer's instructions.

PER2:Luc SCN Organotypic Culture

Brains were removed from pups of at least 10 days of age and sectioned as reported previously [9]. Bioluminescent emissions from PER2::Luc SCN slices were recorded by PMTs for at least 10 days of recording as described previously [9]. Waveforms of rhythmic bioluminescence emission from slices were analyzed in BRASS software (Paul E. Brown and Andrew J. Millar).

Statistics

Statistics for circadian wheel running, all qPCR analysis, IHC and ISH quantification were carried out using the following methods. All statistics were carried out using SPSS v16.0. Student's *t*-tests were used in cases of direct comparison across one categorical variable (e.g. genotype), and one dependent measurement (Type II phase shift, nonparametric circadian wheel running analyses and pupillometry). Multivariate one-way ANOVAs were used in the case of one categorical variable (e.g. genotype or tissue type), and multiple dependent measurements (e.g. activity across multiple light cycles or gene expression across multiple ZT times). The effects of genotype (circadian wheel running activity, SCN qPCR target gene expression, ISH AVP expression, IHC AVP expression and corticosterone plasma concentration) or tissue type (SCN qPCR *Six6* tissue validation) were assessed using pair-wise planned comparisons and these *p*-values are displayed in the figure legends.

A multivariate two-way ANOVA was used to assess qPCR SCN clock gene expression allowing the comparison of two categorical variables (genotype and clock gene) across multiple dependent variables (gene expression at different ZT times). The effects of genotype were assessed using pair-wise planned comparisons and these *p*-values are displayed in the figure legends. Statistical analysis of video-tracking was carried out using repeated measures two-way ANOVAs (between subjects factor of genotype, within subjects factor of ZT time in 10 min bins and dependent variables of distance travelled or immobile time) with main effects of genotype being followed up with planned comparisons and Bonferroni post-hoc analysis of comparisons which were not accounted for in the experimental design. For direct comparison of total activity or immobility, within a given time frame, Student's *t*-tests were performed.

Human Data

Human rest-activity patterns are referred to as part of a larger study with two representative examples one from a non-institutionalised patient fulfilling DSM-IV criteria for schizophrenia and another from a healthy unemployed control (Figure S2) [10]. The patient was stable with respect to psychopathology and social function before entering and during the study. No subject had a history of impaired vision, head injury or significant substance abuse, and the control had no history of any medical condition, including psychiatric illness. Rest-activity and ambient light exposure was monitored simultaneously using a wrist-worn actigraph (Actiwatch-Light, Cambridge Neurotechnology, Ltd) and analysed using CNT Sleep analysis 5.54 software. Recordings were taken from the non-dominant wrist for 6 weeks continuously with a 2-min sampling rate under a self imposed light dark cycle in a home environment. Healthy control: female, 46 years, recording started in August. Patient with disrupted rhythms: male, 34 years, Olanzapine (20mg), recording started in November. All subjects were recruited in West London, United Kingdom. The study protocol was approved by the Ealing and West London Mental Health Trust Local Research Ethics Committee. All participants gave written informed consent.

Supplemental References

1. Nolan, P.M., Peters, J., Strivens, M., Rogers, D., Hagan, J., Spurr, N., Gray, I.C., Vizor, L., Brooker, D., Whitehill, E., et al. (2000). A systematic, genome-wide, phenotype-driven mutagenesis programme for gene function studies in the mouse. Nat Genet *25*, 440-443.
2. Albrecht, U., Zheng, B., Larkin, D., Sun, Z.S., and Lee, C.C. (2001). MPer1 and mper2 are essential for normal resetting of the circadian clock. J Biol Rhythms *16*, 100-104.
3. Grosse, J., and Hastings, M.H. (1996). A role for the circadian clock of the suprachiasmatic nuclei in the interpretation of serial melatonin signals in the Syrian hamster. J Biol Rhythms *11*, 317-324.
4. Van Someren, E.J., Swaab, D.F., Colenda, C.C., Cohen, W., McCall, W.V., and Rosenquist, P.B. (1999). Bright light therapy: improved sensitivity to its effects on rest-activity rhythms in Alzheimer patients by application of nonparametric methods. Chronobiol Int *16*, 505-518.
5. Pallier, P.N., Maywood, E.S., Zheng, Z., Chesham, J.E., Inyushkin, A.N., Dyball, R., Hastings, M.H., and Morton, A.J. (2007). Pharmacological imposition of sleep slows cognitive decline and reverses dysregulation of circadian gene expression in a transgenic mouse model of Huntington's disease. J Neurosci *27*, 7869-7878.
6. Fisher, S.P., Godinho, S.I., Pothecary, C. A., Hankins, M. W., Foster, R. G., and Peirson, S. N. (2011). Rapid assessment of sleep/wake behaviour in mice. J Biol Rhythms *In press*.
7. Jeans, A.F., Oliver, P.L., Johnson, R., Capogna, M., Vikman, J., Molnar, Z., Babbs, A., Partridge, C.J., Salehi, A., Bengtsson, M., et al. (2007). A dominant mutation in Snap25 causes impaired vesicle trafficking, sensorimotor gating, and ataxia in the blind-drunk mouse. Proc Natl Acad Sci U S A *104*, 2431-2436.
8. Selby, C.P., Thompson, C., Schmitz, T.M., Van Gelder, R.N., and Sancar, A. (2000). Functional redundancy of cryptochromes and classical photoreceptors for nonvisual ocular photoreception in mice. Proc Natl Acad Sci U S A *97*, 14697-14702.
9. Maywood, E.S., Reddy, A.B., Wong, G.K., O'Neill, J.S., O'Brien, J.A., McMahon, D.G., Harmar, A.J., Okamura, H., and Hastings, M.H. (2006). Synchronization and maintenance of timekeeping in suprachiasmatic circadian clock cells by neuropeptidergic signaling. Curr Biol *16*, 599-605.
10. Wulff, K., Dijk, D.J., Middleton, B., Foster, R.G., and Joyce, E. (2011). Sleep and circadian dysruption in schizophrenia patients. Br J Psychiatry *in press.*
